# Digital rehabilitation care planning for people with chronic diseases (RehaPro-SERVE): study protocol for a German multicentre randomised controlled trial

**DOI:** 10.1186/s13063-024-08571-2

**Published:** 2024-10-29

**Authors:** Kristina Buch, Veronika van der Wardt, Ulf Seifart, Jörg Haasenritter, Catharina Maulbecker-Armstrong, Pellumbesha Seferi, Annette Becker

**Affiliations:** 1 Department of Primary Care, University of Marburg, Karl-Von-Frisch-Straße 4, Marburg, 35032 Germany; 2 Hospital Sonnenblick, German Pension Insurance, Amöneburger Straße 1-6, Marburg, 35043 Germany; 3 Faculty of Health Sciences, University of Applied Sciences Central Hesse, Wiesenstraße 14, Giessen, 35390 Germany

**Keywords:** Inpatient rehabilitation, Primary health care, Randomised controlled trial, Work incapacity, Process evaluation, Complex intervention, Return to work, Multidisciplinary care planning, Case management, Digital platform

## Abstract

**Background:**

Chronic diseases are a significant and growing problem of our time. They impair the ability to work and increase the risk of early retirement. To support the return to work, rehabilitation services can be applied for in Germany. Currently, the application system for rehabilitation allows only a limited degree of individualisation of the treatment and is associated with a lack of multidisciplinary communication. To facilitate rehabilitation care planning, we developed a complex intervention. A digital, platform-based case management approach (intervention) will ensure multidisciplinary communication and the tailored selection of medical treatments and/or non-medical support measures. The overall objective is to assess the effectiveness of the intervention compared to treatment as usual (control condition). The German Federal Ministry of Labour and Social Affairs (BMAS) funds the RehaPro-SERVE study (grant number: 661R0053K1).

**Methods:**

This is the protocol for an investigator-initiated, pragmatic, multicentre, randomised and controlled two-arm parallel-group superiority trial with embedded qualitative process evaluation. The study will be conducted in Hesse state, Germany. *N* = 59 primary care physicians will be recruited and tasked with the recruitment of six eligible patients each. Eligibility criteria: age 40–60; minimum of 4-week work disability due to musculoskeletal, oncologic or psychological conditions or the post-COVID-19 syndrome within the last 6 months; at high risk for early retirement. In total, *n* = 352 patients will be randomised with a 1:1 allocation to intervention or control group and stratified by primary care practice using permuted blocks. The primary outcome is the number of days of sick leave during a 12-month period after the assumed completion of treatments (t1 to t2). Secondary outcomes include the number of days of sick leave (self-report), work ability, and health-related quality of life, as well as data from the qualitative process evaluation.

**Discussion:**

The results of the study will inform the design of future care services and provide valuable information on multidisciplinary case management in the context of rehabilitation care planning. The results of the qualitative process evaluation will further contribute to the understanding of facilitating and hindering factors.

**Trial registration:**

DRKS-German Clinical Trials Register, DRKS0 00242 07. Registered on 22 March 2021.

**Supplementary Information:**

The online version contains supplementary material available at 10.1186/s13063-024-08571-2.

## Administrative information

The numbers in curly brackets in this protocol refer to SPIRIT checklist item numbers. The order of the items has been modified to group similar items (see http://www.equator-network.org/reporting-guidelines/spirit-2013-statement-defining-standard-protocol-items-for-clinical-trials/)

**Table:** 

Title {1}	Digital rehabilitation care planning for people with chronic diseases (RehaPro-SERVE): study protocol for a German multicentre randomised controlled trial
Trial registration {2a and 2b}.	DRKS-German Clinical Trials Register, DRKS0 00242 07. Registered on 22 March 2021. All items from the World Health Organization Trial Registration Data Set for this protocol can be found in Additional file 1.
Protocol version {3}	Protocol 3.0 04/07/2022
Funding {4}	German Federal Ministry of Labour and Social Affairs (BMAS) (grant number: 661R0053K1). Bundesministerium für Arbeit und Soziales (BMAS) Wilhelmstraße 49 10,117 Berlin Germany (+ 49) 3018 5270 info@bmas.bund.de
Author details {5a}	1 Department of Primary Care, University of Marburg, Karl-von-Frisch-Straße 4, 35,032 Marburg, Germany 2 Hospital Sonnenblick, German Pension Insurance, Amöneburger Straße 1–6, 35,043 Marburg, Germany 3 Faculty of Health Sciences, University of Applied Sciences Central Hesse, Wiesenstraße 14, 35,390 Giessen, Germany
Name and contact information for the trial sponsor {5b}	Deutsche Rentenversicherung Hessen PD Dr. Ulf Seifart Amöneburger Str. 1–6 35,043 Marburg Germany (+ 49) 6421 295 501
Role of sponsor {5c}	The sponsor had no influence on the study design, writing of this article and the decision to submit it for publication.

## Introduction

### Background and rationale {6a}

Reports show that chronic diseases are on the rise in high-income countries [1]. While they have a substantial impact on the quality of life of those affected [2, 3] and are associated with reduced work ability [1], they also represent a risk factor for early retirement [1]. In Germany, rehabilitation offers an opportunity for tertiary prevention and has shown to be effective in supporting patients with oncological, musculoskeletal or mental disorders in their return to work [4–6]. Working-age people can apply for rehabilitation therapies from the German pension insurance, which is usually responsible for approving and providing medical rehabilitation if patients have accumulated at least 5 years of pension contributions and their work ability is reduced or at risk. The application process is initiated by the patients themselves, the attending primary care physician (PCP) or other specialised physician [7, 8]. The PCP usually assists in the preparation and submission of the application and also sends a medical report to the German pension insurance [7, 8]. If personal (e.g. reduced working ability) and insurance law requirements (e.g. insurance period) are met, the application can be approved. Approved rehabilitation can take place on an outpatient or inpatient basis, with inpatient rehabilitation accounting for around 80% in Germany [9]. Inpatient stays usually span 3 weeks and consist of a combination of different treatment elements like medical treatments, exercise therapy, health education, psychological counselling, relaxation techniques, occupational therapy, work-related interventions or socio-legal counselling. Due to the legal requirements and approval of therapies from pension insurance services only, the current system only supports individualisation of the therapy to a very limited extent. However, patients consider a good fit between the design of the rehabilitation therapy and their individual needs an important factor in the evaluation of rehabilitation [10]. Further problems, counteracting an easy access to rehabilitation therapies, are seen in the application and approval process by PCPs. In particular, the process is perceived as time-consuming [7, 11], bureaucratic [7, 11] and non-transparent [11, 12]. Communication deficits and a lack of interdisciplinary coordination are also reported [7, 11, 12]. However, approaches that use multidisciplinary care planning improve functional outcomes for people with chronic or complex care needs [13] and strategies to improve communication across disciplines are associated with improved health and patient satisfaction [14]. Digital solutions, such as platform-based approaches, could provide useful support for care planning and facilitate communication between different stakeholders [15–18].

This paper describes the design of the RehaPro-SERVE study, an investigator-initiated, pragmatic, multicentre, randomised and controlled two-arm parallel-group superiority trial with an embedded process evaluation including an interview study. Through a platform-based case management approach, the study aims to facilitate rehabilitation care planning. A complex intervention [19] was developed that involves multidisciplinary communication and enables the tailored selection of appropriate rehabilitation therapy, medical treatments (MT) or non-medical support measures (NMSM). The digital platform-based solution also ensures communication between stakeholders in an efficient and timely way. The study will compare this new approach to rehabilitation care planning with routine care.

### Objectives {7}

The overall objective is to assess the effectiveness of the developed platform-based case management approach (intervention condition) for rehabilitation care planning compared to treatment/care planning as usual (control condition).

The primary estimand is defined by the following attributes:

- Treatment condition: assignment to platform-based case management approach (intervention condition) for rehabilitation care planning compared to treatment/care planning as usual (control condition) including the effects of withdrawal from rehabilitation care.
- Target population: adults aged 40 to 60 years with a minimum of 4-week (20 workdays) work disability due to musculoskeletal, oncologic or psychological conditions or the post-COVID-19 syndrome (PCS) within the last 6 months prior to study inclusion (patient-reported information) and a high risk of early retirement
- Endpoint: cumulative sick leave days, health insurance data, during the period of 12 months after completion of the treatment (t1 to t2).
- Intercurrent events and strategies to address them: plausible and important intercurrent events include the patients’ withdrawal from the rehabilitation due to any reason, are addressed in the treatment condition attribute and handled with the treatment policy strategy.

The secondary estimands differ from the primary only in regard to the endpoints: self-reported cumulative sick leave days during the period of 12 months after completion of the treatment (t1 to t2); work ability, as measured by the Work Ability Index (WAI, German version) [20] and the health-related quality of life, as measured by the Short Form 36 Health Survey (SF-36, German version) [21, 22] 12 months after completion of the treatment (t2).

We also aim to describe the delivered process in terms of adherence, use of treatments (MT/NMSM), implementation of the intervention, treatments (MT/NMSM) recommended on the digital platform as well as involvement of a social worker. Moreover, facilitating and hindering factors that influence the success of the intervention and to identify adaptations that are necessary for a successful implementation will be investigated qualitatively as a part of the process evaluation.

### Trial design {8}

The RehaPro SERVE Trial is an investigator-initiated, pragmatic, multicentre, randomised and controlled two-arm parallel-group superiority trial. Initially, PCPs will be recruited by the study team, enrolled in the study and tasked with recruiting patients with musculoskeletal, oncological or psychological conditions or PCS. Patients will be randomised using a 1:1 allocation to either the intervention group (rehabilitation care planning with a multidisciplinary platform-based case management approach) or the control group (treatment as usual) stratified by primary care practice and using permuted blocks. A parallel process evaluation will be completed, which will examine adherence, treatment decisions (MT/NMSM), implementation of the intervention, as well as involvement of the social worker. An interview study will be part of the process evaluation and will be conducted with PCPs, patients and other stakeholders involved in the intervention (public health physician, case administrator and social worker), to qualitatively investigate facilitating and hindering factors which influence the success of the intervention and to identify adaptations that are necessary for successful implementation. Reporting of the study will follow the CONSORT guidelines for reporting parallel group randomised trials [23].

## Methods: participants, interventions and outcomes

### Study setting {9}

Recruitment will take place in the setting of primary care in the state of Hesse (Germany). The aim is to recruit 59 PCPs from urban and rural areas in the regions of Frankfurt, Marburg-Biedenkopf as well as central and northern Hesse in the study. Each PCP will be asked to recruit six patients from their practice.

### Eligibility criteria {10}

The inclusion criteria for PCPs are:

- Willingness to recruit six eligible patients
- Willingness to use the digital communication platform for rehabilitation care planning
- Participation in the interview study is optional

There were no specified exclusion criteria for PCPs.

The inclusion criteria for patients are:

- Age 40 to 60 years
- In total a minimum of 4-week (20 workdays) work disability due to musculoskeletal, oncologic or psychological conditions or the PCS within the last 6 months prior to study inclusion (patient-reported information)
- High risk of early retirement (determined with a scores ≤ 36 on the Work Ability Index [20])
- Participation in the interview study is optional

Exclusion criteria for patients are:

- Primary disease addiction or cranial brain trauma
- Ongoing application for rehabilitation treatment
- Being retired, receiving a retirement pension or disability benefits or ongoing application
- Being covered by private health insurance
- Working as a civil servant
- Permanently living abroad
- Inability to speak and read sufficient German to read the study information and complete assessments
- Health issues that prevent participation in rehabilitation therapy

### Who will take informed consent? {26a}

PCPs and eligible patients will be included in the study respectively at a personal appointment with the study team, at which the informed consent will be signed. The materials of the informed consent are presented in Additional file 2 in the original language. For more details on the recruitment procedure see SPIRIT item 15.

### Additional consent provisions for collection and use of participant data and biological specimens {26b}

Possible further follow-up assessments with patients are planned beyond the scope of the study. After taking part in the study, patients are therefore asked for their written consent to be contacted by the German Pension Insurance 5 and 10 years after participating in the study.

## Interventions

### Explanation for the choice of comparators {6b}

As inpatient treatment may not be the best treatment option for all participating patients, the comparator is not the usual rehabilitation application. Treatment-as-usual was chosen as a comparator to enable a comparison with actual care. Patients in the control group will continue to receive usual care by their attending PCP. It will be possible for them to apply for rehabilitation treatments (MT/NMSM) using paper-based forms.

### Intervention description {11a}

Multidisciplinary care planning for patients allocated to the intervention group will follow a digital case management approach, which allows flexible and clinical-based decision-making. In this complex intervention [19], case conferences (CC) will be arranged on the digital communication platform Cankado [24]. Stakeholders from different disciplines and holding different roles will be involved in the CCs:

- PCPs will be experts for their patients, enter them on the digital communication platform and provide for the rehabilitation relevant medical information about them. They will make an initial proposal for a treatment (MT/NMSM) which will be discussed on the platform with the stakeholders. Once the discussion about appropriate treatments (MT/NMSM) reaches consensus, the attending PCP will propose and discuss the treatment option (MT/NMSM) with the patient.
- A case administrator, employed by the German pension insurance, will facilitate the communication between the stakeholders and support cooperation if necessary. If the patient agrees to the treatment proposal (MT/NMSM) from the CC, it will be the case administrator’s task to arrange the appropriate programme.
- A public health physician, also employed by the German pension insurance and experienced in rehabilitation care planning, will also attend the CC and can recommend appropriate measures (MT, inpatient rehabilitation treatments, outpatient therapies) from pension insurance services.
- To offer a different and non-clinical perspective, an employee of the employment agency or jobcentre will also participate in the CC and can suggest appropriate NMSM and more work-related services from their portfolio, like vocational training, requalification or rehabilitation.
- If a specific need is identified by stakeholders in the CC, patients can receive support from a social worker in accessing treatments (MT/NMSM). The social worker is also employed by the German pension insurance and will not be involved in the CC itself.

The digital CC is designed to facilitate rehabilitation care planning for PCPs as well as patients and to improve the quality of care through different components:

- Efficient, multidisciplinary communication of stakeholders. Usage of the digital communication platform Cankado [24] will enable communication without the need for a meeting. A text box offers the option to respond to other stakeholders’ comments and suggestions, which ensures time flexibility and facilitates fast response processes.
- Tailoring and flexibilisation of treatments (MT/NMSM). Care planning for patients can contain regular or innovative treatments (MT/NMSM). Innovative treatments (MT/NMSM) can include services from the employment agencies or job centres that are usually not funded by the German pension insurance. It will be also possible for patients to receive treatments (MT/NMSM), for which they do not meet the requirements in routine care (e.g. insufficient insurance participation period). The multidisciplinary perspective and the possibility for individualisation enable tailored treatment (MT/NMSM) offers based on flexible clinical decisions.
- Possibility for additional support (e.g. assisting the patient in coordinating treatments, appointments or transport or planning absences from the family) through the involvement of a social worker.

**Fig. 1:**
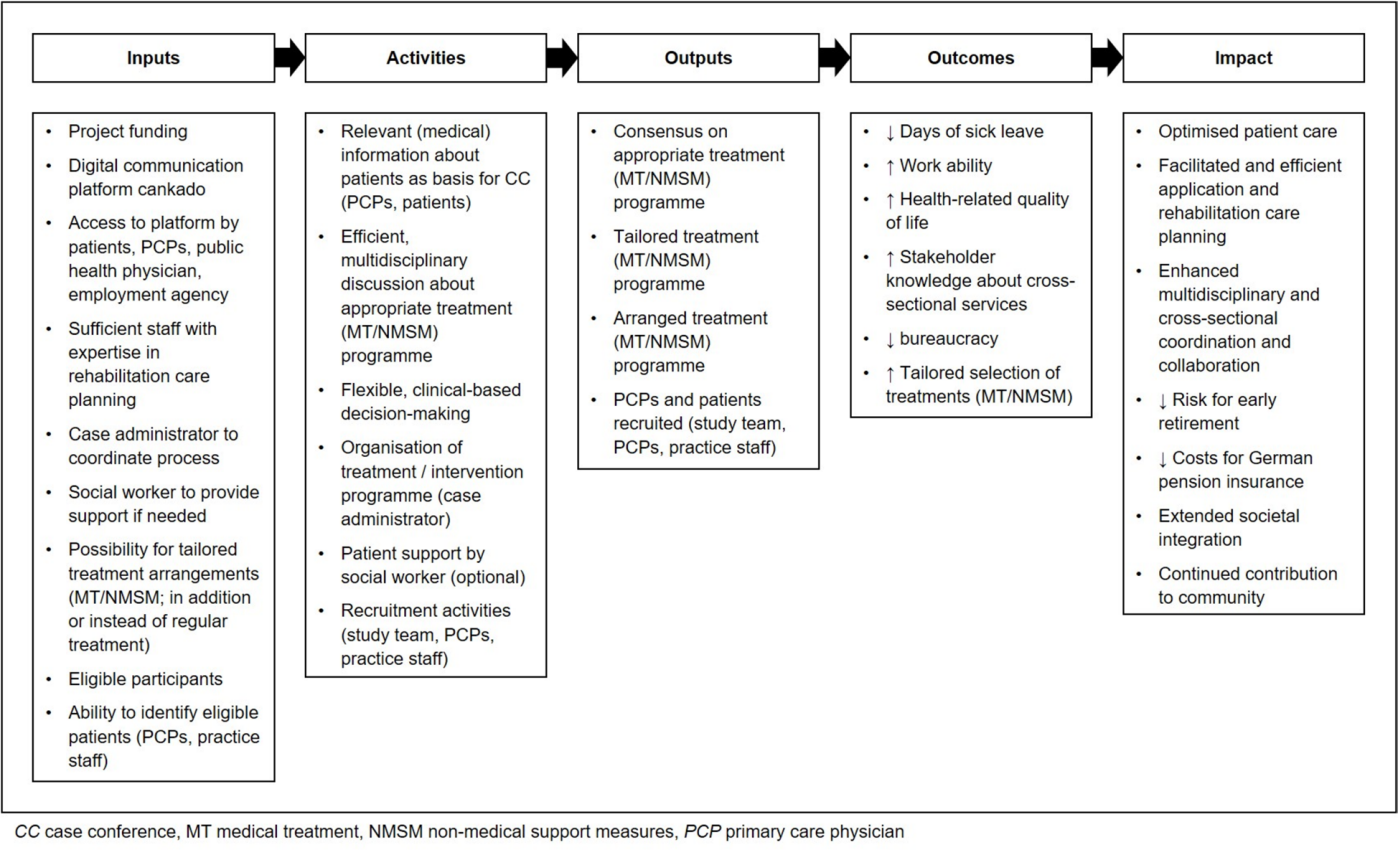
Logic model

An intervention description based on the TIDier checklist [25] is presented in Additional file 3. Also, a logic model was created in keeping with MRC guidance [26] and the W.K. Kellogg Foundation [27], providing a theoretical framework for the intervention (Fig. 1).

### Criteria for discontinuing or modifying allocated interventions {11b}

PCPs as well as patients will be able to withdrawal consent for study participation (e.g. in case of deterioration of health condition) at any time without giving reasons or experiencing any disadvantages. Modifying allocated interventions is not applicable as care planning is meant to be flexible.

### Strategies to improve adherence to interventions {11c}

The case manager will monitor adherence for PCPs in the CC. In the event of difficulties (e.g. delayed processing, missing information on the platform), the study team will be informed and contact the PCP to provide flexible support if required. The case manager also monitors the completeness of the additional questionnaires from patients in the intervention group (see SPIRIT item 18a) on the platform. The social worker can assist patients from the intervention group with all organisational issues regarding the intervention. As part of the monitoring and control process, meetings with the trial steering committee will be held every 14 days to discuss deviations and violations of the protocol and to assess the risk of such (composition of the trial steering committee is explained in SPIRIT item 5d). Adherence to resulting treatment decisions (MT/NMSM) will not be monitored, as the focus of the trial lies on the procedures of the CC. However, if needed, the social worker can be administered to support patients in taking up the offer.

### Relevant concomitant care permitted or prohibited during the trial {11d}

Patients with ongoing application procedures for rehabilitative services cannot participate in the study. Patients from the control group will be able to apply for rehabilitation treatments (MT/NMSM) in the regular way. Apart from this, the study protocol does not restrict access or referral to any usual care services.

### Provisions for post-trial care {30}

The aim of the intervention is to facilitate access to appropriate rehabilitative treatments (MT/NMSM) for patients at high risk of early retirement. After the trial, all participants will return to routine care. If there will be a continuing need for healthcare, this can be discussed with the attending PCP and appropriate steps taken. This will be independent of the intervention given. Post-trial care will not be provided as part of the study.

### Outcomes {12}

The primary outcome is the total numbers of days of sick leave between t1 and t2 (12-month period after the assumed completion of treatment (MT/NMSM)). The time points for assessments are presented in Table 1.

#### Primary outcome

Number of days of sick leave: postal enquiry health insurance (time periods inquired: 6 months before baseline till t0, t0 till t1, t1 till t2). Patients will receive a prepared form and a stamped envelope so that the request can be sent to their health insurance directly after signature. The aggregation method for both groups will be the mean number of sick days between t1 and t2 assessed at t2.

#### Secondary outcomes

- Number of days of sick leave: self-report (time periods inquired: 6 months before baseline till t0, t0 till t1, t1 till t2). The aggregation method for both groups will be the mean number of sick days between t1 and t2 assessed at t2.
- Work ability: assessed using the German version of the WAI [20], which indicates the extent to which an employee is capable of performing his or her job under the individual conditions of their own health and the circumstances of the workplace. WAI scores are ranging from 7 to 49 and can be categorised into excellent (44-49), good (37-43), moderate (28-36) or poor (7-27) work ability. The aggregation method for both groups will be the mean WAI scores at t2.
- Health-related quality of life: measured with the German version of the SF-36 [21, 22], which assesses subjective health on eight dimensions that can be assigned to two sum scales, physical and mental health. SF-36 scores are ranging from 0 to 100, with higher scores indicating better subjective health. We will use the German norm sample from 1994 to determine the sum scales [22]. The aggregation method for both groups will be the mean SF-36 scores at t2.

#### Process evaluation

- Withdrawal rates: withdrawals and reasons will be documented during the course of the study
- Time between baseline and start of first treatment (MT/NMSM, in days): patient questionnaire about start and end dates of treatments (MT/NMSM; t1, t2)
- Treatment (MT/NMSM) recommended on the digital communication platform (only intervention group): recorded on digital communication platform (innovative treatment: yes/no; free text on the final decision)
- Treatments (MT/NMSM) used: patient questionnaire (free text and categorical classification of the treatment; t1, t2)
- Involvement of social worker and reasons for involvement (only intervention group): recorded on digital communication platform (yes/no)
- Duration of CC (in days, only intervention group): data from digital communication platform
- Qualitative Interview data on facilitating and hindering factors that influence the success of the intervention and help to identify adaptations that are necessary for successful implementation.

### Participant timeline {13}

Eligible PCPs will be asked to sign informed consent (Additional file 2) and then complete a short questionnaire containing demographic and practice characteristics. Afterwards, they will start screening for eligible patients. Data will be collected at three time points: baseline (t0), 6 months (t1) and 18 months (t2) during personal or telephone appointments with a research assistant. We expect CCs or other application procedures, waiting times and possible treatments (MT/NMSM) to be completed 6 months after baseline (t1). The time schedule of patients' enrolment, interventions, and assessments is displayed in Table 1.

**Table 1:**
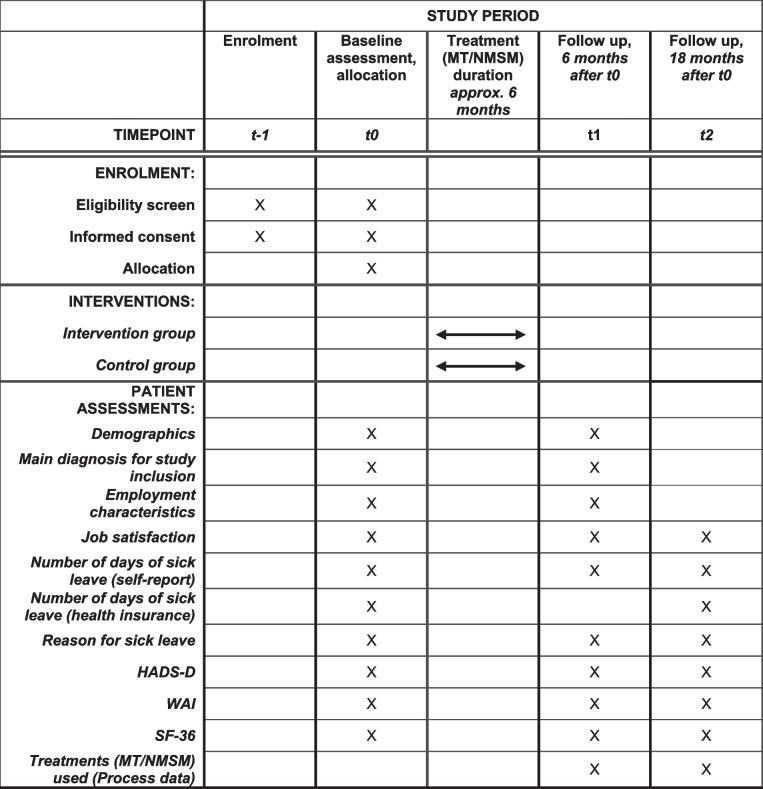
Time schedule of patients’ enrolment, interventions and assessments

*HADS* Hospital anxiety and depression scale, *WAI* Work ability index, *SF-36* Short Form 36 Health Survey, *MT* Medical treatment, *NMSM* Non-medical support measures

Interviews with PCPs will start earliest after closure of the CC of their first patient allocated to the intervention group and are planned to be conducted at various time points in order to provide a broad picture. Interviews with patients will also take place at different time points, but no earlier than after the offer of treatment (MT/NMSM). Other persons involved in the CC (public health physician, case administrator and social worker) will be asked for an interview at the end of the RCT.

### Sample size {14}

Based on a systematic review of workplace interventions to prevent work incapacity [27], a difference of 34 days of incapacity to work (12-month period between t1 (assumed end of treatments) and t2) between the intervention and control group is assumed and applied for sample size calculation. A sample size of 149 patients in each group will have 90% power to detect a difference in means of 34 days of sick leave assuming that the common standard deviation is 90 days using a two-group *t*-test with a 5% two-sided significance level. Assuming that about 15% will be lost to follow-up or withdraw from the study, 352 participants have to be included. If a PCP would recruit six participants, about 59 PCPs must participate in the study. Sample size calculation was performed using R: A Language and Environment for Statistical Computing [28] and the package pwr [29]. The number of patients to be recruited per practice (*n* = 6) is based on feasibility and experience regarding the reasonableness for participating practices.

Participating in the interview study, as a part of the process evaluation, will be optional. The sample size for interviews will depend on data saturation [30]. We plan on interviewing 12 to 25 PCPs and patients each. Persons involved in the CC (public health physician, case administrator, social worker) will also be invited for the interview.

### Recruitment {15}

PCPs will be recruited, using a research practice network, but additional practices will be contacted if needed. Practices will be approached by the study team by post, telephone, email or in person. In case of interest, study information can be sent and a personal information appointment will be arranged with a member of the study team. Prior to signing the consent form PCPs will have the opportunity to ask further questions. Consented PCPs will be asked to fill out the baseline assessment.

Potential patients will be identified by PCPs, via consultation or through the practice database, based on eligibility criteria. PCPs will be reminded to recruit eligible patients by members of the study team on a regular basis. Contact will be in person, by telephone or by mail. If recruitment numbers are low, PCPs will be asked about the difficulties and support that can be offered, e.g. in searching the practice database. PCPs will receive a compensation of 200 euros for study participation, and further 50 euros for each patient recruited. This allows for a maximum compensation of 500 euros. Contact with identified patients can be arranged flexibly by the PCPs and can be made in person by telephone or by post. PCPs will provide information about the study and check the eligibility criteria together with patients. Patients’ self-reports will be used for eligibility-checking. Eligible and interested patients can then contact the study team themselves or provide written consent for their PCP to pass on their contact details to the study team (Additional file 2). Eligibility criteria will be double-checked by the study team, by telephone or in person. Patients who are not eligible will not be able to participate in the study. As the identification and initial approach of potential patients takes place in the primary care practice, the exact number of patients screened will not be determined. For the CONSORT flow chart [23], the number of patients who will have given their written consent to be contacted by the study team will be used. The reasons for exclusion from the study programme, which will be determined by the study team during the double-check, are noted in a list. If interested and eligible, a personal appointment with a member of the study team will take place. The appointment is arranged according to the patient's needs and can be organised at the patient's home, the PCP`s practice or other suitable location. Patients will receive written study information and will have the possibility to ask questions. Once the patient signed informed consent (Additional file 2), the baseline assessment and randomisation will be completed. There will be no financial compensation for participating patients.

## Assignment of interventions: allocation

### Sequence generation {16a}

Eligible and consenting patients will be randomised after baseline assessment with a 1:1 allocation stratified by primary care practice and using permuted blocks of six (as this is the number of patients to be recruited in the practices).

Prior to the commencement of the trial, an algorithm was used to create random sequences and discarded if they were not balanced (1:1) or too trivial, to ensure concealment for as long as possible (till the randomisation of the last patient). One of the random sequences was then randomly assigned to each practice to be recruited. This procedure was carried out by an independent researcher who was not involved in the conduct of the study in any other way.

### Concealment mechanism {16b}

Randomisation will take place using the web-based application randomar [31], programmed by the independent researcher who also generated the randomisation sequence. The allocation of patients is conducted by computer-generated random sequence and is concealed from the study team, patients and attending PCPs until the time of randomisation, at least for the first five patients. For the recruitment of the last patient in a practice, however, the allocation can no longer be concealed. The allocation will be recorded in a database and can no longer be changed. The web-based application will be only accessible by trained members of the study team and protected by a password.

### Implementation {16c}

Once patients consent study participation baseline assessment will take place. Afterwards, a member of the study team will access the web-based randomisation application. The allocation result will be displayed immediately to the study team member and will be logged in a database. Patients will be informed and receive written information about the randomisation result. The attending physician will be informed about the result and the next steps to be taken by post, to ensure data privacy, or in person, if the patient enrolment took place in the practice.

## Assignment of interventions: blinding

### Who will be blinded {17a}

Due to the nature of the study and the required involvement of PCPs, blinding of patients and the study staff directly involved in the trial is not possible. The statistician will be blinded until database lock after (blinded) data review and finalisation of the statistical analysis plan.

### Procedure for unblinding if needed {17b}

Only the trial statistician will be blinded. We do not anticipate a situation where unblinding to the analysis will be needed. In the event the trial statistician is no longer blinded, another statistician will perform the analysis, if feasible.

## Data collection and management

### Plans for assessment and collection of outcomes {18a}

Baseline data for PCPs will be collected on paper, immediately after signing informed consent and during a personal appointment with the study team. Baseline assessments for PCPs will contain demographic (gender, age, work experience, hours of work per week) and practice characteristics (practice size, location (urban/rural) and type (individual/group practice)). The planned time points for collecting patient data are summarised in Table 1, while SPIRIT item 12 provides detailed information on primary and secondary outcomes as well as process evaluation. In addition to outcome data, the following data will be collected from patients at baseline (t0), after 6 months (t1) and 18 months (t2):

- Demographics: gender, age, household size, years of education
- Main diagnosis for study inclusion: musculoskeletal, oncologic or psychological conditions or the PCS
- Employment characteristics: employment status, hours of employment per week
- Job satisfaction: assessed on a 1–10 Likert scale
- Reason for sick leave: musculoskeletal, oncological, psychological or PCS
- Symptoms of anxiety and depression: measured with the German version of the Hospital Anxiety and Depression Scale (HADS-D) [32], which is widely used and internationally accepted as a screening tool for anxiety and depression in people with physical health conditions and disorders. The scores of the two subscales (anxiety and depression) are ranging from 0 to 21, with higher scores indicating more severe symptoms.

All patient data will be collected on paper. Data collection will take place during personal appointments or a telephone call with a member of the study team. In this case, patients will receive the questionnaires in advance by post. The process evaluation will use a mix of data (see SPIRIT item 12).

Patients in the intervention group will answer further questionnaires directly in the digital platform Cankado [24], again the WAI [20], and the Würzburger Screening [33], which helps to decide to what extent a person has occupational problems and needs occupationally oriented and vocational rehabilitation services as well as further medical anamnesis questionnaires. However, these data will be only used for decision-making in the CC and will not be evaluated separately by the study team.

### Plans to promote participant retention and complete follow-up {18b}

Patients will be contacted by the study team (by telephone or mail) before each data collection point, to encourage retention and the completion of follow-ups. Contact will be arranged flexibly by telephone and/or mail according to the preference of the patient. The importance of data collection for the success of the study will be emphasised in both groups in order to motivate patients to continue participation at follow-up. Data collection will be completed by a research assistant. Appointments will be arranged flexibly and can take place by telephone or at a preferred location (e.g. the primary care practice or the patient’s home), to make it as easy as possible for patients to participate in data collection. As part of the process evaluation, the reasons for and numbers of participant dropouts will be monitored.

### Data management {19}

All data, except for SF-36 [21, 22], data collected by the health insurance companies and the dates of data collection are entered using the FormPro software for automatic recognition and recording of completed questionnaires [34] and double-checked by a study assistant. All other data is entered manually by a study assistant and double-checked. Regarding the query management, the study team will take care that the questionnaires are completed correctly during data collection. Discrepancies that will arrive nevertheless during paper-based data collection will be discussed by the study team and, if necessary, declared as missing. The data preparation will be documented in the dataset. A data management plan has been developed based on the European General Data Protection Regulation (GDPR) 2018. Only the research team at the University of Marburg will have access to the collected data. Personal data will be stored on the server at the University of Marburg, password-protected, and separate from the research data as well as in a locked closet in the office of the research team. Personal data will be deleted 10 years after the end of the study. The collection of research data via the Cankado communication platform [24] also follows the GDPR and data will be stored on a server in Germany. During the CCs, no personal data, except for the questionnaires that patients answer on the platform, will be made available to the attendants. This data will be used to make appropriate treatment (MT/NMSM) recommendations in the context of the CC and will not be stored by the study team. All other research-relevant data from Cankado [24] will be exported anonymously after the study is completed and stored password-protected on the server at the University of Marburg. The data will be stored separately from the other research data collected. There will be no linkage between the regular research data and the data collected by Cankado [24]. The interviews will be recorded and transcribed verbatim. Interview data will be pseudonymised. The transcription will be carried out either by the study team itself or by an external transcription agency. The recordings of the interviews and the final transcription will be stored on the server of the University of Marburg.

### Confidentiality {27}

All data collected will be handled confidentially and in accordance with the consent provided by participants. For further information on data management see SPIRIT item 19.

### Plans for collection, laboratory evaluation and storage of biological specimens for genetic or molecular analysis in this trial/future use {33}

N/a This trial will not involve collection, laboratory evaluation, and storage of biological specimens.

## Statistical methods

### Statistical methods for primary and secondary outcomes {20a}

The participant flow will be reported according to the CONSORT guideline [23], which will show withdrawals and drop-outs. PCP and patient characteristics as well as outcomes, and process evaluation data will be descriptively reported using mean values and standard deviations for normally distributed data and median and range for not normally distributed data. Categorical data will be presented in amount and percentage.

All analyses will be performed using a modified intention-to-treat approach on both primary and secondary outcomes. According to a treatment policy estimand strategy, all participants without missing outcome data will be analysed according to their randomised treatment assignment. However, we will exclude cases with missing data due to dropout or loss to follow-up from the main analyses (for details, see item 20c).

For the primary outcome, we will fit a linear regression model with the cumulative sum of sick leave days during the period between t1 and t2, reported by health insurance, as a dependent variable and the treatment indicator as an independent variable, adjusted for sex, age, baseline number of sick leave days and baseline WAI [20] to control for differences between groups at baseline despite randomisation. Additionally, PCP IDs will be included as random intercepts in order to adjust for influence from stratified randomisation [35]. We will report the primary outcome as adjusted between-group mean differences with a 95% confidence interval. We will conduct analogue analyses for the secondary outcomes of self-reported sick leave days, work ability (WAI, 20) and health-related quality of life (SF36, 21, 22).

Interview data will be transcribed and qualitatively analysed using thematic analysis [36].

### Interim analyses {21b}

N/a. No formal interim analyses are planned.

### Methods for additional analyses (e.g. subgroup analyses) {20b}

Subgroup analysis will be performed for the intervention group based on regression models according to age, gender and main diagnosis for study inclusion to determine treatment effect heterogeneity.

### Methods in analysis to handle protocol non-adherence and any statistical methods to handle missing data {20c}

According to a treatment policy estimand strategy [37], all participants without missing outcome data will be analysed according to their randomised treatment assignment. This also applies to participants for whom one of the following post-randomisation, intercurrent events occurs which could also occur in daily clinical practice: study participants of both groups may decide for personal reasons not to take up or to withdraw from the rehabilitation programme offered. It is also possible that a change in their state of health would not allow them to undergo rehabilitation. However, it could not occur that a participant randomised to the control group switches to the intervention group, since this intervention is currently not available in clinical practice, e.g. outside the study setting.

In the main analyses, we will exclude cases with missing data on the variables considered in the analysis of the primary and secondary outcomes due to dropout or loss to follow-up. If available, we will report the reasons for missingness. We will provide a table showing the distribution of baseline characteristics stratified by intervention group for all participants being randomised and for all participants being analysed. We will impute the missing values using multiple imputation and provide sensitivity analyses for primary and secondary outcomes using the imputed data [38].

### Plans to give access to the full protocol, participant-level data and statistical code {31c}

The full protocol and the resulting data set (individual participant data for the principle analysis) will be made available to other researchers upon reasonable request.

## Oversight and monitoring

### Composition of the coordinating centre and trial steering committee {5d}

The direct study team will consist of the scientific staff and study assistants. The study team will run the trial day-to-day and provide organisational support. The study team will meet at least on a weekly basis. The trial statistician will not be a member of the study team. The trial steering committee will consist of the members of the study team, the chief investigator, the overall study coordinators, the case administrator, public health physician and social worker. Meetings will take place every second week to monitor the conduct and progress of the study.

### Composition of the data monitoring committee, its role and reporting structure {21a}

A data monitoring committee will not be established. However, data monitoring will be carried out by the scientific staff of the study team and the case manager will monitor adherence to the protocol in the CCs. If there is any uncertainty, issues will be discussed in the weekly study team meetings and if necessary in the biweekly meetings of the trial steering committee.

### Adverse event reporting and harms {22}

The intervention (CC) is not expected to lead to adverse events or harms. If significant adverse events occur due to the digital case management, the intervention will be stopped. Participants will not be asked specifically about potential harms. There will be an opportunity for participants in the interview study to report on potential harms through observations and experiences.

### Frequency and plans for auditing trial conduct {23}

No auditing is planned for trial conduct.

### Plans for communicating important protocol amendments to relevant parties (e.g. trial participants, ethical committees) {25}

Changes to the protocol require approval from the funder as well as the Ethics Committee prior to implementation. Participating PCPs and (if relevant) patients will then be notified by the study team. If necessary, the study materials will be adapted.

## Dissemination plans {31a}

After the data analysis, the research group will publish the results in an open access medical journal.

## Discussion

This paper describes the study protocol of a randomised controlled trial comparing a new approach to rehabilitation care planning with routine care for people with musculoskeletal, oncological or psychological conditions or PCS. The first three conditions are among the most frequent indications for medical rehabilitation in Germany [39], making them a relevant target group. The study will investigate the effectiveness of an innovative, digital solution to rehabilitation care or other intervention planning using a case management approach. The complex intervention [19] was designed to facilitate the application and access processes to rehabilitation or other appropriate treatments (MT/NMSM), while enabling multidisciplinary communication and tailored clinical decision-making.

Due to the planned inclusion of *n* = 59 PCP practices from rural and urban regions, the results can be assumed to be highly transferable to other primary care settings in Germany. To ensure the feasibility and acceptability of the study procedures and materials, these were tested in advance in a small-scale feasibility study (recruitment between November 2021 and August 2022) [40]. It was shown that, similar to other studies [41, 42], a challenge will be to recruit patients through the practices. Therefore, the inclusion criteria have already been adjusted compared to the feasibility phase. The time and staff required for recruitment for this RCT is expected to be high, but is nevertheless considered feasible.

A limitation is the randomisation technique stratified by primary care practice and using permuted blocks of six (as this is the number of patients to be recruited in the practices). By this design, allocation concealment will not be given for the last (sixth) patient recruited in a practice. This increases the risk of selection bias [43]. Another limitation of the chosen randomisation method is that not all PCPs may be able to recruit six patients. In this case, the equal distribution of patients to both groups would no longer be given. It can also be argued that spill-over effects may occur, as the focus of participating PCPs may have a greater emphasis on the rehabilitation care and intervention planning than without the study. This may also accelerate or initiate processes in the control group. Also, if the study finds differences between the groups, this would be even more indicative of an intervention effect. However, a spill-over effect could also hide the true effect (Type II error). In order to reduce spill-over effects, the use of cluster randomisation was also discussed. Ensuring the chronology of such randomisation (first recruitment, then randomisation) did not appear feasible in the context of the study (long recruitment periods lead to longer waiting times for patients). Without adherence to this chronology, there would be no allocation concealment at all and the risk of recruitment bias would be increased [44]. Therefore, we decided on the stratified randomisation. Also, an equal distribution of workload for PCPs is given.

Another limitation is, that there will be no control of whether the in the CC recommended treatments (MT/NMSM) are actually used. It should be noted that the intervention is particularly about the application and decision-making processes and the offer of treatments (MT/NMSM). Similarly, only these can be influenced within the framework of the study. However, when a person can actually participate in a rehabilitation or other intervention depends on a number of further factors, such as the availability of rehabilitation care slots.

The results of the study will inform the design of future care provision and provide valuable information about multidisciplinary and cross-sectional collaboration. Findings from the embedded process evaluation and included interview study will further contribute to understanding factors that hinder or facilitate the success of the intervention and to identify any changes that are needed for successful implementation.

## Trial status

Protocol version 3.0 04/07/2022. Recruitment began in September 2022 and was planned to conclude in January 2024. Due to the low recruitment numbers, recruitment for the RCT was terminated early in December 2023. (Recruitment for the feasibility study was conducted between November 2021 and August 2022.)

## Supplementary Information

Additional file 1. All items from the World Health Organization Trial Registration Data Set for this protocol

Additional file 2. Informed Consent Materials

Additional file 3. Intervention description based on TIDieR checklist

## Data Availability

N/a. Access to study material is not planned.

## Abbreviations

BMAS: “Bundesministerium für Arbeit und Soziales” German Federal Ministry of Labour and Social Affairs
CC: Case conference
DRKS: “Deutsches Register Klinischer Studien “ German Clinical Trials Register
GDPR (European): General Data Protection Regulation
HADS-D: Hospital Anxiety and Depression Scale, German version
MT: Medical treatment
NMSM: Non-medical support measures
PCP: Primary Care Physician
PCS: Post-COVID-Syndrome
SF-36: Short Form 36 Health Survey
SPIRIT: Standard Protocol Items: Recommendations for Interventional Trials
WAI: Work Ability Index
