# Supplementary material for: Digital rehabilitation care planning for people with chronic diseases (RehaPro-SERVE): study protocol for a German multicentre randomised controlled trial
Source: Trials. 2024 Oct 29;25:728. doi: 10.1186/s13063-024-08571-2 (PMC11520684; doi:10.1186/s13063-024-08571-2)
Supplement: Supplementary file 1 — Additional file 1. All items from the World Health Organization Trial Registration Data Set for this protocol [file 13063_2024_8571_MOESM1_ESM.docx]

**Additional File 1:**

**All items from the World Health Organization Trial Registration Data Set for this protocol**

**Article**: Digital rehabilitation care planning for people with chronic diseases (RehaPro-SERVE): study protocol for a German multicentre randomised controlled trial

**Authors**: Kristina Buch, Veronika van der Wardt, Ulf Seifart, Jörg Haasenritter, Catharina Maulbecker-Armstrong, Pellumbesha Seferi, Annette Becker

| Category | Information |
| --- | --- |
| Primary Registry and Trial Identifying Number | DRKS-German Clinical Trials Register, DRKS0 00242 07. |
| Date of Registration | Registered on 22 March 2021. |
| Funder | Bundesministerium für Arbeit und Soziales (BMAS)  Wilhelmstraße 49  10117 Berlin  Germany |
| Sponsor | Deutsche Rentenversicherung Hessen  PD Dr. Ulf Seifart  Amöneburger Str. 1-6  35043 Marburg  Germany  (+49) 6421 295 501 |
| Contact for Public Queries | Philipps-Universität Marburg, Institut für Allgemeinmedizin  Kristina Buch  Karl-von-Frisch-Straße 4  35043 Marburg  Germany  (+49) 6421 28 25192  kristina.buch@uni-marburg.de |
| Contact for Scientific Queries | Philipps-Universität Marburg, Institut für Allgemeinmedizin  Kristina Buch  Karl-von-Frisch-Straße 4  35043 Marburg  Germany  (+49) 6421 28 25192  kristina.buch@uni-marburg.de |
| Public and Scientific Title | Intersectoral preventive identification, counselling and support of statutorily insured persons with special occupational problems SERVE |
| Scientific Title | Intersectoral preventive identification, counselling and support of statutorily insured persons with special occupational problems SERVE |
| Recruitment Countries | Germany |
| Health Condition(s) or Problem(s) Studied | musculoskeletal, oncological or psychological diseases, post-COVID-19 syndrome |
| Intervention(s) | Arm 1: The cases of the patients in the intervention group are discussed in a virtual case conference by general practitioners, public health physicians and employees of the job center/employment agency. Support with individualised, needs-based recommendations is developed and offered to the patients.  Arm 2: Patients in the control group will be cared for by the general practitioner as usual without the virtual case conference (treatment-as-usual). |
| Inclusion criteria | Age 40-60; In total 4 weeks of incapacity to work in the last 6 month due to musculoskeletal, oncological or psychological diseases or post-COVID-19 syndrome; High risk of permanent incapacity to work (moderate or critical work ability as measured by the Work Ability Index (≤ 36 points)). |
| Exclusion criteria | Primary diagnosis of an addiction disorder or traumatic brain injury, current application for rehabilitation; an old-age or reduced earning capacity pension is being drawn or a corresponding application has been made; civil servant or person equivalent to a civil servant with pension entitlements; persons with private health insurance; persons permanently retired from working life; habitual residence abroad; limited ability to communicate in German (unable to understand the study and/or complete questionnaires); acute illnesses, which make participation in the rehabilitation measures impossible |
| Study Type | Interventional, randomised, controlled trial with two parallel groups |
| Date of First Enrollment | 19.11.2021 |
| Sample size | Target Sample Size: 352  Final Sample Size: 20 |
| Recruitment Status | Recruiting stopped (after recruiting started) |
| Primary Outcome | A reduction in the cumulative incapacity to work within one year after the expected completion of treatment (< 34 days) in the intervention group compared to the control group (health insurance data). |
| Secondary Outcomes | 1. A reduction in the cumulative incapacity to work within one year after the expected completion of treatment in the intervention group compared to the control group (patient self-report).  2. An increase in work ability as measured by the Work Ability Index in the intervention group compared to the control group one year after the expected completion of treatments.  3. An improvement in health-related quality of life as measured by the SF-36 in the intervention group compared to the control group one year after the expected completion of treatments.  4. An evaluation of the implementation of the intervention and the mechanisms of effectiveness (process evaluation with qualitative interviews). |
| Ethics Review | The Ethics Committee from the Faculty of Medicine Ethics Committee at the University of Marburg granted approval for the study on 1 March 2021 (reference number 164/20). |
| Completion date | 11.12.2023 (End of recruitment) |
| IPD sharing statement | Plan to share IPD: Yes  Individual participant data for the principle analysis will be made available to other researchers upon reasonable request. |
