# Supplementary material for: Digital rehabilitation care planning for people with chronic diseases (RehaPro-SERVE): study protocol for a German multicentre randomised controlled trial
Source: Trials. 2024 Oct 29;25:728. doi: 10.1186/s13063-024-08571-2 (PMC11520684; doi:10.1186/s13063-024-08571-2)
Supplement: Supplementary file 2 — Additional file 2. Informed Consent Materials [file 13063_2024_8571_MOESM2_ESM.pdf]

Zum Verbleib bei Hausarzt/Hausärztin

Philipps-Universität Marburg
Institut für Allgemeinmedizin
Studie SERVE
Tel.: 06421 2825194
Mobil: 0151-50838220
Fax: 06421 2865121
E-Mail: serve2020@uni-marburg.de
Anschrift: Karl-von-Frisch-Str. 4
35043 Marburg
Web: http://www.uni-marburg.de/fb20/allgprmed

Studie SERVE

Studieninformation für Hausärztinnen/Hausärzte: Studienteilnahme
(Randomisierte Studie)

Sehr geehrte Dame, sehr geehrter Herr,

wir bitten Sie, an der Studie SERVE „Sektorenübergreifende präventive Identifikation, Beratung und Unterstützung von Versicherten mit besonderen beruflichen Problemlagen“ teilzunehmen. In dieser Studie werden Patientinnen und Patienten, die ein Risiko tragen perspektivisch einen Antrag auf Erwerbsminderungsrente zu stellen, frühzeitig Maßnahmen angeboten, die an den individuellen Bedarf angepasst sind und damit schnell und effektiv Hilfestellungen geben sollen. Es wird untersucht, ob hierdurch langfristig Erwerbsunfähigkeiten aus gesundheitlichen Gründen verringert werden können.

An dieser Studie beteiligt sind die Deutsche Rentenversicherung Hessen, die Philipps-Universität Marburg, die Technische Hochschule Mittelhessen sowie die Arbeitsagenturen und Jobcenter Marburg und Frankfurt. Das Programm wird gefördert vom Bundesministerium für Arbeit und Soziales. Für den Projektteil, um den es hier geht, ist die Abteilung für Allgemeinmedizin der Philipps Universität Marburg zuständig.

Studienablauf

Während der Studie bitten wir Sie, sechs Patientinnen oder Patienten zu kontaktieren, die folgende Kriterien erfüllen: Alter 40-60 Jahre, Arbeitsunfähigkeit mind. 4 Wochen in den letzten 6 Monaten aufgrund einer muskuloskelettalen, psychischen, onkologischen Erkrankung oder Long-Covid-19 Syndrom. Außerdem bitten wir Sie, einen Kurzfragebogen zu Ihrer Person und Praxis auszufüllen.

Hat eine Patientin/ein Patient Interesse an der Teilnahme, wird der Kontakt zum Studienteam der Abteilung für Allgemeinmedizin hegestellt. Wir beantworten Fragen zur Studie und prüfen die Studieneignung. Die Patientin/der Patient wird dann zufällig einer von zwei Gruppen zugeteilt („randomisiert“). Die Versorgung der Patienten/innen der einen Gruppe (Interventionsgruppe)

werden über eine digitale Plattform („Sozialmedizinisches Kolloquium") von Hausärztinnen/Hausärzten, Sozialmedizinern/innen und Mitarbeitenden von Jobcenter/Arbeitsagentur besprochen. Hierbei wird ein Unterstützungskonzept mit entsprechenden individuellen, bedarfsgerechten Empfehlungen erarbeitet. Diese Maßnahmen können z.B. schneller und flexibler verordnet werden. Die andere Gruppe (Kontrollgruppe) wird wie gewohnt weiterhin ohne digitale Fallbesprechung durch den Hausarzt/die Hausärztin betreut (Treatment-as-usual). Auch für Patienten/Patientinnen in der Kontrollgruppe können z.B. Leistungen zur Teilhabe am Arbeitsleben oder eine medizinische Rehabilitation beantragt werden, hier findet die Betreuung allerdings nur durch den Hausarzt/die Hausärztin statt. Die genauen Abläufe erläutern wir Ihnen gern bei einem Besuch in Ihrer Praxis.

Einige Hausärztinnen und Hausärzte werden wir bitten, in einem Interview über die Arbeit mit dem Sozialmedizinischen Kolloquium (SMK) und ihre Einschätzung zu dessen Nutzen zu berichten. Die Teilnahme am Interview ist optional, die Einwilligung zum Interview erfolgt separat.

<u>Datenschutz</u>

Alle personenbezogenen und versorgungsrelevanten Daten unterliegen dem Datenschutz und der Schweigepflicht, welcher das gesamte Studienpersonal unterliegt. Die Forschungsdaten (z.B. Fragebögen) werden pseudonymisiert, d.h. mit einer Codierung und versehen, sodass eine Zuordnung zu Ihrer Person nicht mehr möglich ist. Personenbezogene Daten und Forschungsdaten werden getrennt und passwortgeschützt aufbewahrt. Ihre personenbezogenen Daten werden nach Studienende (April 2025) gelöscht.

<u>Aufwandsentschädigung</u>

Für die Teilnahme an der Studie erhalten Sie zunächst eine Aufwandsentschädigung in Höhe von 200€ für den Erstaufwand plus 50€ pro rekrutiertem Patient/rekrutierter Patientin zu Studienende im Jahr 2025.

Vielen Dank für Ihr Interesse an dieser Studie!

Ergänzende Information für Studienteilnehmer gemäß Europäischer Datenschutz- Grundverordnung (DSGVO)

Rechtsgrundlage

Die Rechtsgrundlage zur Verarbeitung der Sie betreffenden personenbezogenen Daten bilden Ihre freiwillige Einwilligung gemäß DS-GVO sowie der Deklaration von Helsinki (Erklärung des Weltärztebundes zu den ethischen Grundsätzen für die medizinische Forschung am Menschen) und der Leitlinie für Gute Klinische Praxis.

Bezüglich Ihrer Daten haben Sie folgende Rechte (Artikel 13 ff. DS-GVO):

Recht auf Auskunft
Sie haben das Recht auf Auskunft über die Sie betreffenden personenbezogenen Daten, die im Rahmen der klinischen Studie erhoben, verarbeitet oder ggf. an Dritte übermittelt werden (Aushändigen einer *kostenfreien* Kopie) (Artikel 15 DS-GVO).

Recht auf Berichtigung
Sie haben das Recht Sie betreffende unrichtigen personenbezogene Daten berichtigen zu lassen (Artikel 16 und 19 DS-GVO).

Recht auf Löschung
Sie haben das Recht auf Löschung Sie betreffender personenbezogener Daten, z. B. wenn diese Daten für den Zweck, für den sie erhoben wurden, nicht mehr notwendig sind (Artikel 17 und 19 DS-GVO).

Recht auf Einschränkung der Verarbeitung
Unter bestimmten Voraussetzungen haben Sie das Recht auf Einschränkung der Verarbeitung zu verlangen, d.h. die Daten dürfen nur gespeichert, nicht verarbeitet werden. Dies müssen Sie beantragen. Wenden Sie sich hierzu bitte an Ihren Prüfer oder an den Datenschutzbeauftragten des Prüfzentrums (Artikel 18 und 19 DS-GVO).

Im Falle der Berichtigung, Löschung, Einschränkung der Verarbeitung werden zudem all jene benachrichtigt, die Ihre Daten haben (Artikel 17 (2) und Artikel 19 DS-GVO).

Recht auf Datenübertragbarkeit
Sie haben das Recht, die Sie betreffenden personenbezogenen Daten, die Sie dem Verantwortlichen für die klinische Studie / klinische Prüfung bereitgestellt haben, zu erhalten. Damit können Sie beantragen, dass diese Daten entweder Ihnen oder, soweit technisch möglich, einer anderen von Ihnen benannten Stelle übermittelt werden (Artikel 20 DS-GVO).

Zum Verbleib bei Hausarzt/Hausärztin

Widerspruchsrecht
Sie haben das Recht, jederzeit gegen konkrete Entscheidungen oder Maßnahmen zur Verarbeitung der Sie betreffenden personenbezogenen Daten Widerspruch einzulegen (Art 21 DSGVO). Eine solche Verarbeitung findet anschließend grundsätzlich nicht mehr statt.

Einwilligung zur Verarbeitung personenbezogener Daten und Recht auf Widerruf dieser Einwilligung
Die Verarbeitung Ihrer personenbezogenen Daten ist nur mit Ihrer Einwilligung rechtmäßig (Artikel 6 DS-GVO).

Sie haben das Recht, Ihre Einwilligung zur Verarbeitung personenbezogener Daten jederzeit zu widerrufen. Es dürfen jedoch die bis zu diesem Zeitpunkt erhobenen Daten durch die in der Patienteninformation- und Einwilligungserklärung zu der jeweiligen klinischen Studie / Prüfung genannten Stellen verarbeitet werden (Artikel 7, Absatz 3 DSGVO).

Benachrichtigung bei Verletzung des Schutzes personenbezogener Daten („Datenschutzpannen")
Hat eine Verletzung des Schutzes personenbezogener Daten voraussichtlich ein hohes Risiko für Ihre persönlichen Rechte und Freiheiten zur Folge, so werden Sie unverzüglich benachrichtigt (Artikel 34 DSGVO).

Möchten Sie eines dieser Rechte in Anspruch nehmen, wenden Sie sich bitte an die unten genannte für die Datenverarbeitung zuständige Person. Außerdem haben Sie das **Recht, Beschwerde bei der/den Aufsichtsbehörde/n einzulegen,** wenn Sie der Ansicht sind, dass die Verarbeitung der Sie betreffenden personenbezogenen Daten gegen die DS-GVO verstößt (**siehe Kontaktdaten**).

<u>Kontaktdaten:</u>

	**Für die Datenverarbeitung Verantwortliche/r**	**Datenschutzbeauftragte/r**	**Datenschutz-Aufsichtsbehörde**
Name:	Prof. Dr. Annette Becker<br>Philipps Universität Marburg<br>Abteilung für Allgemeinmedizin, Präventive und Rehabilitative Medizin	Datenschutzbeauftragter der Philipps-Universität Marburg	Der Hessische Datenschutzbeauftragte
Adresse:	Karl-von-Frisch-Str. 4<br>35043 Marburg	Biegenstraße 10<br>35032 Marburg<br>(Paketpost: 35037 Marburg)	Gustav-Stresemann-Ring 1<br>65189 Wiesbaden
Telefon:	06421- 28 26120	06421-2826155	Telefon: 0611-140 80
E-Mail:	allgemeinmedizin@uni-marburg.de	datenschutz@uni-marburg.de	poststelle@datenschutz.hessen.de

Zurück ins Studienzentrum

Philipps-Universität Marburg

Institut für Allgemeinmedizin

Studie SERVE

Tel.: 06421 2825194

Mobil: 0151-50838220

Fax: 06421 2865121

E-Mail: serve2020@uni-marburg.de

Anschrift: Karl-von-Frisch-Str. 4
35043 Marburg

Web: http://www.uni-marburg.de/fb20/allgprmed

Studie SERVE

Einwilligungserklärung zur Studienteilnahme für Hausärztinnen/Hausärzte

(Randomisierte Studie)

Ich, Frau/Herr ___________________________________ (**Namen bitte deutlich schreiben!**)

wurde heute vollständig über Inhalt und Bedeutung der Studie „SERVE - Sektorenübergreifende präventive Identifikation, Beratung und Unterstützung von Versicherten mit besonderen beruflichen Problemlagen" aufgeklärt. Ich habe die Studieninformation gelesen und verstanden. Ich hatte die Möglichkeit und ausreichend Zeit, Fragen zu stellen und habe die Antworten verstanden.

Ich weiß, dass ich jederzeit und ohne Angabe von Gründen meine Einwilligung zur Teilnahme an dieser Studie widerrufen kann, ohne dass mir daraus Nachteile erwachsen.

Ich bin mit der im Rahmen der Studie erfolgenden Aufzeichnung meiner personenbezogenen Daten und ihrer Weitergabe entsprechend den datenschutzrechtlichen Ausführungen in der Studieninformation einverstanden.

Ich erkläre mich hiermit einverstanden, an der Studie „SERVE" teilzunehmen.

Eine Kopie der Studieninformation und dieser Einwilligungserklärung habe ich erhalten.

_______________ _______________________________

Ort, Datum **Unterschrift Studienteilnehmer/in**

Ich bestätige, dass ich oben genannten Studienteilnehmer/oben genannten Studienteilnehmerin bezüglich Zweck, Nutzen und Art der Studie aufgeklärt habe.

_______________ _______________________________

Ort, Datum **Unterschrift Aufklärer/in**

Zurück ins Studienzentrum

Philipps-Universität Marburg

Institut für Allgemeinmedizin

Studie SERVE

Tel.: 06421 2825194

Mobil: 0151-50838220

Fax: 06421 2865121

E-Mail: serve2020@uni-marburg.de

Anschrift: Karl-von-Frisch-Str. 4
35043 Marburg

Web: http://www.uni-marburg.de/fb20/allgprmed

Studie SERVE

Einwilligungserklärung für Patientinnen/Patienten zur Weitergabe von Kontaktdaten

Ich, Frau/Herr ___________________________________ (Namen bitte deutlich schreiben!) wurde unverbindlich über Studie SERVE „Sektorenübergreifende präventive Identifikation, Beratung und Unterstützung von Versicherten mit besonderen beruflichen Problemlagen“ der informiert und habe Interesse, weitere Informationen zu erhalten.

Ich bin damit einverstanden, dass meine Kontaktdaten an das Studienteam der Universität Marburg weitergegeben werden, damit eine Mitarbeiterin der Abteilung für Allgemeinmedizin Kontakt zu mir aufnehmen kann. In einem persönlichen Telefonat wird mir die Studie nochmals ausführlich erklärt und es können offene Fragen geklärt werden.

Ich bin damit einverstanden, dass meine Kontaktdaten bis zur Kontaktaufnahme passwortgeschützt gespeichert werden. Im Fall, dass ich nicht an der Studie teilnehme, werden meine Kontaktdaten sofort und endgültig gelöscht.

Mir ist bewusst, dass diese Einverständniserklärung nur die Weitergabe meiner Kontaktdaten an das Projektteam der Abteilung für Allgemeinmedizin beinhaltet. Falls ich nach Erhalt weiterer Informationen, nach Beantwortung aller meiner Fragen und nach Bedenkzeit an der Studie teilnehmen möchte, muss eine weitere Einverständniserklärung zur Teilnahme an der Studie unterschrieben werden.

Zurück ins Studienzentrum

Meine Kontaktdaten sind:

Name: ____

Adresse: ____

Telefonnummer: ____

E-Mail: ____

Ich weiß, dass ich ohne Angabe von Gründen meine Einwilligung zur Kontaktaufnahme widerrufen kann, ohne dass mir daraus Nachteile entstehen.

Eine Kopie des Informationsblattes und dieser Einverständniserklärung zur Weitergabe von Kontaktdaten habe ich erhalten.

________________ ________________________________

Ort, Datum **Unterschrift Interessent/in**

Ich bestätige, dass ich oben genannten Studienteilnehmer/oben genannten Studienteilnehmerin bezüglich Zweck, Nutzen und Art der Studie aufgeklärt habe.

________________ ________________________________

Ort, Datum **Unterschrift Aufklärer/in der Hausarztpraxis**

Philipps-Universität Marburg

Institut für Allgemeinmedizin

Studie SERVE

Tel.: 06421 2825194

Mobil: 0151-50838220

Fax: 06421 2865121

E-Mail: serve2020@uni-marburg.de

Anschrift: Karl-von-Frisch-Str. 4
35043 Marburg

Web: http://www.uni-marburg.de/fb20/allgprmed

Studie SERVE

Studieninformation für Patientinnen/Patienten: Studienteilnahme

(Randomisierte Studie)

Sehr geehrte Dame, sehr geehrter Herr,

wir bitten Sie, an der Studie SERVE „Sektorenübergreifende präventive Identifikation, Beratung und Unterstützung von Versicherten mit besonderen beruflichen Problemlagen“ teilzunehmen. Ihr Hausarzt/Ihre Hausärztin nimmt an der Studie SERVE teil und schlägt vor, dass Sie ebenfalls teilnehmen. In diesem Projekt werden frühzeitig Rehabilitationsmaßnahmen angeboten, die an den individuellen Bedarf angepasst sind und damit schnell und effektiv Hilfestellungen geben sollen. Es wird untersucht, ob hierdurch langfristig Erwerbsunfähigkeiten aus gesundheitlichen Gründen verringert werden können.

An dieser Studie beteiligt sind die Deutsche Rentenversicherung Hessen, die Philipps-Universität Marburg, die Technische Hochschule Mittelhessen sowie die Arbeitsagenturen und Jobcenter Marburg und Frankfurt. Das Programm wird gefördert vom Bundesministerium für Arbeit und Soziales. Für den Projektteil, um den es hier geht, ist die Abteilung für Allgemeinmedizin der Philipps Universität Marburg zuständig.

Wie läuft die Studie ab?

An der Studie teilnehmen können Patientinnen und Patienten im Alter von 40-60 Jahren, die in den letzten 6 Monaten insgesamt mindestens 4 Wochen wegen einer Muskel-Skelett-Erkrankung, einer psychischen Beeinträchtigung, onkologischen Erkrankung, oder Long-Covid-19 Syndrom arbeitsunfähig sind.

Alle teilnehmenden Patientinnen und Patienten werden zufällig einer von zwei Gruppe zugeordnet. Ihr Hausarzt/Ihre Hausärztin und die Studiengruppe haben keinen Einfluss darauf, welcher Gruppe Sie zugeteilt werden.

Werden Sie der sogenannten „Interventionsgruppe“ zugeteilt, erarbeitet Ihr Hausarzt/Ihre Hausärztin in einer virtuellen Besprechung (dem sogenannten „Sozialmedizinischen Kolloquium“) gemeinsam mit Sozialmedizinern und Mitarbeitenden von Arbeitsamt/Jobcenter ein Behandlungs-

konzept. Dieses Behandlungskonzept wird an Sie individuell angepasst und die Maßnahmen können schneller und flexibler gestaltet werden als in der normalen Versorgung. Ihre Hausärztin/Ihr Hausarzt bleibt dabei immer Ihre Ansprechpartnerin/Ihr Ansprechpartner und wird Ihnen diese Maßnahmen anbieten. Werden Sie der sogenannten „Kontrollgruppe" zugeteilt, betreut Ihr Hausarzt Sie normal weiter. Ihre Hausärztin/Ihr Hausarzt kann dann auch ohne eine virtuelle Besprechung eine Leistung zur Teilhabe am Arbeitsleben oder medizinische Rehabilitation vorschlagen und beantragen.

Zu Beginn der Teilnahme, nach 6 Monaten und nach 18 Monaten werden wir Sie bitten, Fragebögen zu Ihrer Person, Ihrer Arbeit und Ihrer Gesundheit zu beantworten. Zusätzlich würden wir Sie bitten, Ihre Arbeitsunfähigkeitszeiten von Ihrer Krankenversicherung mittels eines vorgefertigten Formulars zu erfragen. Dieses werden Sie zusammen mit einem Rückumschlag von uns erhalten.

Einige Patientinnen und Patienten werden wir nach der Teilnahme an der Rehabilitationsmaßnahme bitten, in einem Interview über Ihre Erfahrungen zu berichten. Die Teilnahme am Interview ist optional, die Einwilligung zum Interview erfolgt separat.

Gegebenenfalls treten wir später nochmal an Sie heran mit der Bitte, weitere Daten erheben zu dürfen. Die Teilnahme daran ist freiwillig und unabhängig von der Studienteilnahme.

Datenschutz

Alle personenbezogenen und versorgungsrelevanten Daten unterliegen dem Datenschutz und der Schweigepflicht, welcher das gesamte Studienpersonal unterliegt. Das Behandlungskonzept der Patientinnen und Patienten, welche der „Interventionsgruppe" zugeteilt werden, wird im Sozialmedizinischen Kolloquium erarbeitet. Die Mitglieder des Sozialmedizinischen Kolloquiums (Hausarzt/Hausärztin, Sozialmediziner/Mitarbeitende der Deutschen Rentenversicherung Hessen, Mitarbeitende von Jobcenter/Arbeitsagentur) müssen hierfür auf personenbezogene Daten (z.B. Name, Geburtsdatum, Kontaktdaten), versorgungsrelevante Daten (z.B. Vorerkrankungen) und Rentenversicherungsdaten zugreifen können. Mit der Einwilligung zur Studienteilnahme stimmen Sie dem zu. Des Weiteren beinhaltet die Einwilligung zur Studienteilnahme die Entbindung der Mitglieder des Sozialmedizinischen Kolloquiums von der ärztlichen Schweigepflicht gegenüber den anderen Mitgliedern des Sozialmedizinischen Kolloquiums.

Die Forschungsdaten (z.B. Fragebögen, Audiodaten, Interviewtranskripte) werden pseudonymisiert, das heißt die Datensätze werden mit einer Kodierung und nicht mit Ihrem Namen versehen, so dass eine Zuordnung zu Ihrer Person nicht mehr möglich ist. Personenbezogene Daten und Forschungsdaten werden getrennt und passwortgeschützt aufbewahrt. Ihre personenbezogenen Daten werden 10 Jahre nach Studienende (Oktober 2035) gelöscht.

Wie geht es jetzt weiter?

Können Sie sich vorstellen teilzunehmen? Wir bitten Sie, uns als Studienteam der Abteilung für Allgemeinmedizin an der Universität Marburg telefonisch, per Fax oder E-Mail zu kontaktieren. Alternativ können Sie ihrem Hausarzt/Ihrer Hausärztin schriftlich erlauben, Ihre Kontaktdaten an uns weiterzugeben, sodass wir uns bei Ihnen melden können. Es handelt sich dabei zunächst einmal um eine unverbindliche Interessensbekundung.
In einem persönlichen Telefonat erklären wir Ihnen den Studienablauf gern noch einmal ausführlich und beantworten alle Ihre Fragen. Dann entscheiden Sie, ob Sie an der Studie teilnehmen möchten.

Sie erreichen uns unter folgenden Kontaktdaten:
Tel.: 06421 2825192
Fax: 06421 2865121
E-Mail: serve2020@uni-marburg.de

Wir danken Ihnen für Ihr Interesse und laden Sie ein, an der Studie teilzunehmen!

Ergänzende Information für Studienteilnehmer gemäß Europäischer Datenschutz- Grundverordnung (DSGVO)

Rechtsgrundlage

Die Rechtsgrundlage zur Verarbeitung der Sie betreffenden personenbezogenen Daten bilden Ihre freiwillige Einwilligung gemäß DS-GVO sowie der Deklaration von Helsinki (Erklärung des Weltärztebundes zu den ethischen Grundsätzen für die medizinische Forschung am Menschen) und der Leitlinie für Gute Klinische Praxis.

Bezüglich Ihrer Daten haben Sie folgende Rechte (Artikel 13 ff. DS-GVO):

Recht auf Auskunft
Sie haben das Recht auf Auskunft über die Sie betreffenden personenbezogenen Daten, die im Rahmen der klinischen Studie erhoben, verarbeitet oder ggf. an Dritte übermittelt werden (Aushändigen einer *kostenfreien* Kopie) (Artikel 15 DS-GVO).

Recht auf Berichtigung
Sie haben das Recht Sie betreffende unrichtigen personenbezogene Daten berichtigen zu lassen (Artikel 16 und 19 DS-GVO).

Recht auf Löschung
Sie haben das Recht auf Löschung Sie betreffender personenbezogener Daten, z. B. wenn diese Daten für den Zweck, für den sie erhoben wurden, nicht mehr notwendig sind (Artikel 17 und 19 DS-GVO).

Recht auf Einschränkung der Verarbeitung
Unter bestimmten Voraussetzungen haben Sie das Recht auf Einschränkung der Verarbeitung zu verlangen, d.h. die Daten dürfen nur gespeichert, nicht verarbeitet werden. Dies müssen Sie beantragen. Wenden Sie sich hierzu bitte an Ihren Prüfer oder an den Datenschutzbeauftragten des Prüfzentrums (Artikel 18 und 19 DS-GVO).

Im Falle der Berichtigung, Löschung, Einschränkung der Verarbeitung werden zudem all jene benachrichtigt, die Ihre Daten haben (Artikel 17 (2) und Artikel 19 DS-GVO).

Recht auf Datenübertragbarkeit
Sie haben das Recht, die Sie betreffenden personenbezogenen Daten, die Sie dem Verantwortlichen für die klinische Studie / klinische Prüfung bereitgestellt haben, zu erhalten. Damit können Sie beantragen, dass diese Daten entweder Ihnen oder, soweit technisch möglich, einer anderen von Ihnen benannten Stelle übermittelt werden (Artikel 20 DS-GVO).

Widerspruchsrecht
Sie haben das Recht, jederzeit gegen konkrete Entscheidungen oder Maßnahmen zur Verarbeitung der Sie betreffenden personenbezogenen Daten Widerspruch einzulegen (Art 21 DSGVO). Eine solche Verarbeitung findet anschließend grundsätzlich nicht mehr statt.

Einwilligung zur Verarbeitung personenbezogener Daten und Recht auf Widerruf dieser Einwilligung
Die Verarbeitung Ihrer personenbezogenen Daten ist nur mit Ihrer Einwilligung rechtmäßig (Artikel 6 DS-GVO).

Sie haben das Recht, Ihre Einwilligung zur Verarbeitung personenbezogener Daten jederzeit zu widerrufen. Es dürfen jedoch die bis zu diesem Zeitpunkt erhobenen Daten durch die in der Patienteninformation- und Einwilligungserklärung zu der jeweiligen klinischen Studie / Prüfung genannten Stellen verarbeitet werden (Artikel 7, Absatz 3 DSGVO).

Benachrichtigung bei Verletzung des Schutzes personenbezogener Daten („Datenschutzpannen“)
Hat eine Verletzung des Schutzes personenbezogener Daten voraussichtlich ein hohes Risiko für Ihre persönlichen Rechte und Freiheiten zur Folge, so werden Sie unverzüglich benachrichtigt (Artikel 34 DSGVO).

Möchten Sie eines dieser Rechte in Anspruch nehmen, wenden Sie sich bitte an die unten genannte für die Datenverarbeitung zuständige Person. Außerdem haben Sie das **Recht, Beschwerde bei der/den Aufsichtsbehörde/n einzulegen,** wenn Sie der Ansicht sind, dass die Verarbeitung der Sie betreffenden personenbezogenen Daten gegen die DS-GVO verstößt (**siehe Kontaktdaten**).

Kontaktdaten:

	**Für die Datenverarbeitung Verantwortliche/r**	**Datenschutzbeauftragte/r**	**Datenschutz-Aufsichtsbehörde**
Name:	Prof. Dr. Annette Becker<br>Philipps Universität Marburg<br>Abteilung für Allgemeinmedizin, Präventive und Rehabilitative Medizin	Datenschutzbeauftragter der Philipps-Universität Marburg	Der Hessische Datenschutzbeauftragte
Adresse:	Karl-von-Frisch-Str. 4<br>35043 Marburg	Biegenstraße 10<br>35032 Marburg<br>(Paketpost: 35037 Marburg)	Gustav-Stresemann-Ring 1<br>65189 Wiesbaden
Telefon:	06421- 28 26120	06421-2826155	Telefon: 0611-140 80
E-Mail:	allgemeinmedizin@uni-marburg.de	datenschutz@uni-marburg.de	poststelle@datenschutz.hessen.de

Zurück ins Studienzentrum

Philipps-Universität Marburg

Institut für Allgemeinmedizin

Studie SERVE

Tel.: 06421 2825194

Mobil: 0151-50838220

Fax: 06421 2865121

E-Mail: serve2020@uni-marburg.de

Anschrift: Karl-von-Frisch-Str. 4
35043 Marburg

Web: http://www.uni-marburg.de/fb20/allgprmed

Studie SERVE

Einwilligungserklärung zur Studienteilnahme für Patientinnen/Patienten

(Randomisierte Studie)

Ich, Frau/Herr ___________________________________ (**Namen bitte deutlich schreiben!**)

wurde heute vollständig über Inhalt und Bedeutung der Studie „SERVE - Sektorenübergreifende präventive Identifikation, Beratung und Unterstützung von Versicherten mit besonderen beruflichen Problemlagen“ aufgeklärt. Ich habe die Studieninformation gelesen und verstanden. Ich hatte die Möglichkeit und ausreichend Zeit, Fragen zu stellen und habe die Antworten verstanden.

Ich weiß, dass ich jederzeit und ohne Angabe von Gründen meine Einwilligung zur Teilnahme an dieser Studie widerrufen kann, ohne dass mir daraus Nachteile erwachsen.

Ich bin mit der im Rahmen der Studie erfolgenden Aufzeichnung meiner personenbezogenen Daten und ihrer Weitergabe entsprechend den datenschutzrechtlichen Ausführungen in der Patienteninformation einverstanden.

Ich erkläre mich hiermit einverstanden, an der Studie „SERVE“ teilzunehmen.

Eine Kopie der Studieninformation und dieser Einwilligungserklärung habe ich erhalten.

_______________ _______________________________

Ort, Datum **Unterschrift Studienteilnehmer/in**

Ich bestätige, dass ich oben genannten Studienteilnehmer/oben genannten Studienteilnehmerin bezüglich Zweck, Nutzen und Art der Studie aufgeklärt habe.

_______________ _______________________________

Ort, Datum **Unterschrift Aufklärer/in**
