# Supplementary material for: Digital rehabilitation care planning for people with chronic diseases (RehaPro-SERVE): study protocol for a German multicentre randomised controlled trial
Source: Trials. 2024 Oct 29;25:728. doi: 10.1186/s13063-024-08571-2 (PMC11520684; doi:10.1186/s13063-024-08571-2)
Supplement: Supplementary file 3 — Additional file 3. Intervention description based on TIDieR checklist [file 13063_2024_8571_MOESM3_ESM.docx]

**Additional File 3:**

**Intervention description based on TIDieR checklist^[[1]](#footnote-1)^**

**Article**: Digital rehabilitation care planning for people with chronic diseases (RehaPro-SERVE): study protocol for a German multicentre randomised controlled trial

**Authors**: Kristina Buch, Veronika van der Wardt, Ulf Seifart, Jörg Haasenritter, Catharina Maulbecker-Armstrong, Pellumbesha Seferi, Annette Becker

| Name | RehaPro-SERVE – digital case management platform |
| --- | --- |
| Why? (rationale) | The digital case management platform is designed to facilitate rehabilitation care planning for primary care physicians (PCPs) as well as patients and to improve the quality of care through different components:  1) efficient multidisciplinary discussion and consensus building of stakeholders (PCP, a public health physician and an employee of the employment agency/jobcentre) about appropriate treatment programme, consisting of medical treatments (MT) or non-medical support measures (NMSM). The process will be supported by a case administrator, who is also in charge for the arrangement of the decided programme. Usage of the digital platform will enable communication without the need for a meeting. Communication in writing will further ensure time flexibility and independence as well as fast response processes.  2) tailoring and flexibilisation of treatments (MT/NMSM). Care planning for patients can contain regular or innovative treatments (MT/NMSM). Innovative treatments can include services from the employment agencies or job centres that are usually not funded by the German pension insurance (e.g. occupational therapy, return-to-work support or work-related educational courses). It will be also possible for patients to receive treatments (MT/NMSM), for which they do not meet the requirements in routine care (e.g. insufficient insurance participation period). The multidisciplinary perspective and the possibility for individualisation will enable tailor-made treatment offers (MT/NMSM) based on flexible clinical decisions. Treatments (MT/NMSM) may be arranged on an in- and/or out-patient basis.  3) optional patient support by a social worker, when an additional need for support is seen by physicians. |
| What? (procedure) | PCPs will enter patients on the digital communication platform and add relevant medical information about them. They will make an initial proposal for an appropriate treatment (MT/NMSM) programme, which will be the base for the case conference (CC). Also, all attendants of the CC will be notified and will be able to access the information. Afterwards the discussion will take place in writing, until consensus on an appropriate treatment (MT/NMSM) programme is reached. This proposal will be discussed between the attending PCP and the patient.  If the patient agrees, the treatment (MT/NMSM) programme will be arranged by the case administrator. Support in accessing treatment (MT/NMSM) can be offered from a social worker, if a specific need is identified by the attendants of the CC. Support will be aligned with the needs of the patient and may include assisting the patient in coordinating treatments, appointments or transport or planning absences from the family. |
| Who provided? | - PCPs will be experts for their patients, enter them on the digital platform and provide relevant medical information about them. They will make an initial proposal for treatment (MT/NMSM) which will be discussed afterwards. Once the discussion reaches consensus, the attending PCP will discuss the treatment (MT/NMSM) option with the patient. - A case administrator, employed by the German pension insurance, will facilitate the communication between the stakeholders and support cooperation if necessary. If a patient agrees to the treatment (MT/NMSM) proposal from the CC, it will be the case administrator’s task to arrange the appropriate programme. - A public health physician, also employed by the German pension insurance and experienced in rehabilitation care planning, will also attend the CC and can recommend treatments (MT/NMSM) from pension insurance services. - To offer a different and non-clinical perspective, an employee of the employment agency or jobcentre will also participate in the CC and can suggest appropriate NMSM, as work-related services from their portfolio, like vocational training, requalification or rehabilitation. - If a specific need is identified by stakeholders in the CC, patients can receive support from a social worker in accessing treatment (MT/NMSM). The social worker is also employed by the German pension insurance and will not be involved in the CC itself. |
| How? | Communication on the digital platform will be in writing. The PCP will provide relevant medical information, which can be accessed electronically by the attendants of the CC. After the PCP has made an initial treatment (MT/NMSM) recommendation, the discussion will take place until consensus is reached. |
| Where? | CCs will take place on the digital communication platform Cankado. |
| When and how much? | The CC will begin with the PCP entering the patient on the platform and will end as soon as the discussion has reached a consensus. The CC can be reconvened until the treatment (MT/NMSM) is considered completed by the physicians. The frequency of communication and CCs will be flexible and individually tailored to the case. |
| Tailoring | Based on flexible clinical decision-making, treatment (MT/NMSM) recommendations will be tailored to the individual needs of patients. |
| How well? (adherence/fidelity) | The process evaluation will investigate the delivered process in terms of adherence, use of treatments (MT/NMSM), implementation of the intervention, treatments (MT/NMSM) recommended on the digital platform as well as involvement of the social worker. |

1. Hoffmann T C, Glasziou P P, Boutron I, Milne R, Perera R, Moher D et al. Better reporting of interventions: template for intervention description and replication (TIDieR) checklist and guide BMJ 2014; 348 :g1687 doi:10.1136/bmj.g1687 [↑](#footnote-ref-1)
